# Selection of an Aptamer against the Enzyme 1-deoxy-D-xylulose-5-phosphate Reductoisomerase from *Plasmodium falciparum*

**DOI:** 10.3390/pharmaceutics14112515

**Published:** 2022-11-19

**Authors:** Carlota Roca, Yunuen Avalos-Padilla, Beatriz Prieto-Simón, Valentín Iglesias, Miriam Ramírez, Santiago Imperial, Xavier Fernàndez-Busquets

**Affiliations:** 1 Barcelona Institute for Global Health (ISGlobal), Hospital Clínic, Universitat de Barcelona, Rosselló 149-153, 08036 Barcelona, Spain; 2 Nanomalaria Group, Institute for Bioengineering of Catalonia (IBEC), The Barcelona Institute of Science and Technology, Baldiri Reixac 10-12, 08028 Barcelona, Spain; 3 Nanoscience and Nanotechnology Institute (IN2UB), University of Barcelona, Martí i Franquès 1, 08028 Barcelona, Spain; 4 Department of Biochemistry and Molecular Biomedicine, University of Barcelona, Avda. Diagonal 643, 08028 Barcelona, Spain; 5 Department of Electronic Engineering, Universitat Rovira i Virgili, 43007 Tarragona, Spain; 6 ICREA, Pg. Lluís Companys 23, 08010 Barcelona, Spain; 7 Institut de Biotecnologia i Biomedicina and Departament de Bioquímica i Biologia Molecular, Universitat Autònoma de Barcelona, 08193 Bellaterra, Spain

**Keywords:** *Plasmodium*, DNA aptamers, methyl erythritol phosphate pathway, 1-deoxy-D-xylulose-5-phosphate reductoisomerase

## Abstract

The methyl erythritol phosphate (MEP) pathway of isoprenoid biosynthesis is essential for malaria parasites and also for several human pathogenic bacteria, thus representing an interesting target for future antimalarials and antibiotics and for diagnostic strategies. We have developed a DNA aptamer (D10) against *Plasmodium falciparum* 1-deoxy-D-xylulose-5-phosphate reductoisomerase (DXR), the second enzyme of this metabolic route. D10 binds in vitro to recombinant DXR from *P. falciparum* and *Escherichia coli*, showing at 10 µM a ca. 50% inhibition of the bacterial enzyme. In silico docking analysis indicates that D10 associates with DXR in solvent-exposed regions outside the active center pocket. According to fluorescence confocal microscopy data, this aptamer specifically targets in *P. falciparum* in vitro cultures the apicoplast organelle where the MEP pathway is localized and is, therefore, a highly specific marker of red blood cells parasitized by *Plasmodium* vs. naïve erythrocytes. D10 is also selective for the detection of MEP+ bacteria (e.g., *E. coli* and *Pseudomonas aeruginosa*) vs. those lacking DXR (e.g., *Enterococcus faecalis*). Based on these results, we discuss the potential of DNA aptamers in the development of ligands that can outcompete the performance of the well-established antibody technology for future therapeutic and diagnostic approaches.

## 1. Introduction

Globally, in 2020 there were an estimated 241 million malaria cases, up from 227 million in 2019, and 627,000 malaria deaths were reported, increasing the total number by 12% compared to the previous year [1]. Malaria is mainly present in low-income countries where limited accessibility to treatments as well as poor practices in mosquito bite prevention have led to the prevalence of the disease despite the efforts devoted to controlling it. Malaria is caused by protozoans of the *Plasmodium* genus, which are transmitted to people through the bite of infected female *Anopheles* mosquitoes [2]. Five species of *Plasmodium* can infect humans: *P. vivax, P. ovale, P. malariae, P. knowlesi,* and *P. falciparum*.

The malaria parasite harbors an apicoplast, a vestigial plastid present in the Apicomplexa phylum. There are four pathways that are essential in blood-stage *Plasmodium* parasites, which localize to the apicoplast lumen, namely, DNA replication, protein translation, isoprenoid biosynthesis, and iron-sulfur cluster biosynthesis [3]. Several investigations have shown that the apicoplast is essential for *P. falciparum* survival and therefore have identified it as an interesting drug target [4,5]. For instance, interference in the process of apicoplast segregation during parasite multiplication inside the red blood cell (RBC) produced merozoite stages which, despite being viable, failed to divide in the subsequent growth cycles [6,7]. Parasites exhibiting this delayed death phenotype transmit defective apicoplasts to their daughter cells, which are therefore unable to produce the isoprenoid precursor isopentenyl pyrophosphate (IPP), a metabolic product of the apicoplast which is essential for *Plasmodium* blood stages. Among other effects, IPP depletion causes disruption of protein prenylation and the ensuing cellular trafficking impairment, which decreases the pathogen’s viability [8]. In contrast to delayed death caused by the drugs interfering with the housekeeping processes within the apicoplast, drugs targeting the biosynthesis of fatty acids, isoprenoids, and heme result in rapid growth arrest and death of *P. falciparum* [9,10,11,12].

**Figure 1:**
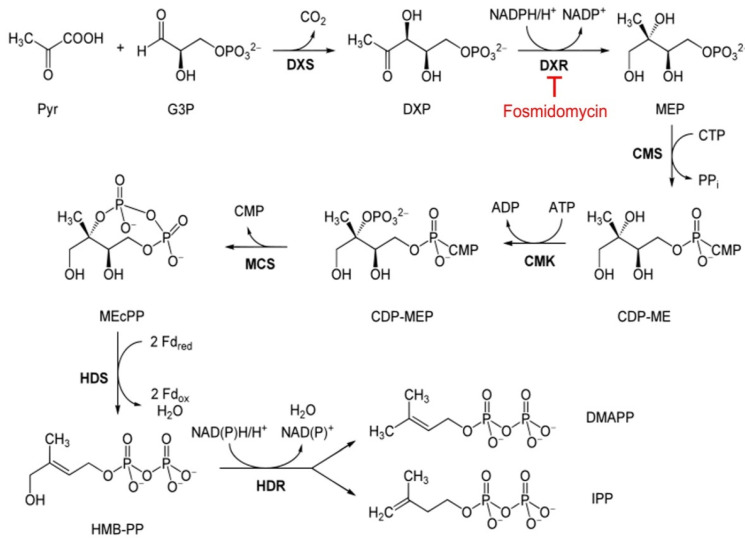
MEP pathway in *P. falciparum*. Abbreviations: Pyruvate (Pyr), glyceraldehyde 3-phosphate (G3P), 1-deoxy-D-xylulose 5-phosphate (DXP), DXP synthase (DXS), DXP reductoisomerase (DXR), 2-C-methyl-D-erythritol 4-phosphate (MEP), MEP cytidylyltransferase (CMS), 4-diphosphocytidyl-2-C-methylerythritol (CDP-ME), CDP-ME kinase (CMK), CDP-ME 2-phosphate (CDP-MEP), 2-C-methyl-D-erythritol 2,4-cyclodiphosphate (MEcPP), MEcPP synthase (MCS), (*E*)-4-hydroxy-3-methyl-but-2-enyl pyrophosphate (HMB-PP), HMB-PP synthase (HDS), HMB-PP reductase (HDR), dimethylallyl pyrophosphate (DMAPP), isopentenyl pyrophosphate (IPP). Adapted from the scheme of Giménez-Oya et al. [21], with permission. 2010, John Wiley and Sons.

Isoprenoids are a chemically diverse group of compounds made up of repeated units of IPP or its isomer dimethylallyl diphosphate (DMAPP). They are present in all organisms, where they play essential roles as enzyme prosthetic groups and also as precursors to ubiquinones and dolichols, which are involved in the electron transfer system in the mitochondrion, protein prenylation and the formation of glycosylphosphatidyl inositol anchors of membrane proteins [13,14,15]. In most eukaryotes and archaea, IPP is synthesized through the cytosolic mevalonate pathway [16], which starts with the condensation of acetyl-CoA and acetoacetyl-CoA. However, in *Plasmodium*, the apicoplast is the sole site of isoprenoid precursor synthesis through the 2-C-methyl-D-erythritol-4-phosphate (MEP) pathway [17]. This metabolic route (Figure 1) has seven enzymatic steps, starting with the formation of 1-deoxy-D-xylulose-5-phosphate (DXP) by condensation of pyruvate and glyceraldehyde-3-phosphate. The subsequent reaction is catalyzed by 1-deoxy-D-xylulose-5-phosphate reductoisomerase (DXR) and involves an intramolecular rearrangement that proceeds through retro-aldol/aldol isomerization of the DXP ketone to the branched aldehyde intermediate 2-C-methyl-D-erythrose-4-phosphate, followed by NADPH-dependent reduction to form MEP [17,18,19]. All the enzymes metabolizing these and the remaining five steps of this metabolic route are not present in vertebrates, pointing to the MEP pathway as an ideal target for the development of new antibiotics and antimalarials [14,20].

DXR is present in all *Plasmodium* stages, and it appears to be essential for the intra-erythrocytic development of the pathogen. The antibiotic fosmidomycin, a DXR inhibitor, is used as treatment against the clinical symptoms of malaria associated with the multiplication of the parasite inside the RBC [22,23]. A close derivative of fosmidomycin, FR-900098, inhibited the in vitro growth of *P. falciparum* with approximately twice the efficacy of the parental compound, showing no evidence of acute toxicity and genotoxicity [24]. The greatest challenge in malaria management is the resistance of the pathogen to conventional monochemotherapies and even to combination therapies, the most effective currently used antimalarial treatment [25]. Due to this resistance rapidly acquired by *Plasmodium* to all available drugs [26], strategies for the continuous rapid identification of new antimalarial agents are urgently required.

DNA aptamers have been developed against many relevant therapeutic targets for a variety of diseases (e.g., cancer, macular degeneration, diabetes, or infectious diseases) [27,28]. There are several advantages of aptamers as biomolecular ligands, such as their small size, remarkable flexibility in engineering their structures, high-temperature stability, animal-free and cost-effective production, and high affinity and selectivity for their targets, which make them attractive alternatives to monoclonal antibodies for a wide range of applications, including biosensor technologies, in vitro diagnostics, biomarker discovery, and therapeutics [29,30]. The first report on *Plasmodium*-specific aptamers dates from 2009 when Barfod et al. successfully isolated a specific RNA aptamer against the DBL1α region of the *P. falciparum* erythrocyte membrane protein 1, which is associated with heme metabolism and parasitized RBC (pRBC) adhesion to blood vessels and erythrocytes [31]. In the same year, Niles et al. isolated a DNA aptamer that specifically bound the heme group and inhibited hemozoin formation in vitro in a similar way as the antimalarial compound chloroquine acts. Consistently loading this aptamer into RBCs resulted in a reduction of *Plasmodium* viability due to the accumulation of toxic heme in the pRBCs [32]. DNA aptamers have also been proposed as sensing elements in future diagnostic devices for the detection of malaria parasites in clinical samples [33,34].

Here, we have applied the systematic evolution of ligands by exponential enrichment (SELEX) method [35,36] to identify a DNA aptamer against *P. falciparum* DXR and have characterized its potential as an apicoplast biomarker and as a component of new diagnostic and therapeutic approaches against malaria.

## 2. Materials and Methods

Unless otherwise indicated, oligonucleotides and other reagents were purchased from the Sigma-Aldrich Corporation (St. Louis, MO, USA).

### 2.1. Preparation of Recombinant Enzymes

A truncated version of the *P. falciparum* DXR (*Pf*DXRt) lacking the sequence corresponding to the apicoplast signal peptide [37] was obtained following an adaptation of a previously described method [38]. Briefly, a codon-optimized gene encoding *Pf*DXRt (UniProt Q8IKG4) was synthetically produced (GenScript Biotech B.V., Leiden, The Netherlands) and cloned into the pGS21a expression vector (GenScript Biotech B.V.; Supplementary Figure S1). The generated pGS21a-*Pf*DXRt plasmid was used to obtain a recombinant enzyme as a fusion protein containing a glutathione-S-transferase (GST) tag at the N-terminus, and 6× His-tags at both ends (6His-GST-*Pf*DXRt-6His), to enhance protein expression and facilitate affinity purification, respectively. *Escherichia coli* BL21 cells were transformed with the pGS21a-*Pf*DXRt vector and grown in Luria-Bertani (LB) medium supplemented with 0.1 mg/mL ampicillin at 37 °C until reaching an OD_600_ of 0.5. After that, expression of 6His-GST-*Pf*DXRt-6His was induced by adding 0.5 mM isopropyl β-D-1-thiogalactopyranoside (IPTG). The bacteria were incubated overnight (15 °C, 180 rpm), harvested by centrifugation (21,500× *g*, 15 min), and resuspended in a lysis buffer: 300 mM NaCl, 1 mM EDTA, 1 mM MgCl_2_, 0.2 mg/mL lysozyme, 20 µg/mL DNAse, 1 mM Pefabloc^®^ SC protease inhibitor (Merck KGaA, Darmstadt, Germany), 50 mM tris-HCl, pH 8.0, supplemented with cOmplete^TM^, Mini, EDTA-free Protease Inhibitor Cocktail (Merck KGaA). The cell suspension was disrupted by sonication by applying pulses of 10 s-on/20 s-off at 80% amplitude on ice to minimize enzyme inactivation. Finally, extracts were centrifuged (43,700× *g*, 4 °C, 30 min), and the distribution of the recombinant protein in the soluble and insoluble fractions was analyzed by sodium dodecyl sulfate-polyacrylamide gel electrophoresis (SDS-PAGE) and Western blot (see below). The 6His-GST-*Pf*DXRt-6His was recovered almost completely in the insoluble fraction, which, to solubilize the recombinant enzyme, was incubated for 30 min in a solubilization buffer (2 M urea, 0.5 M NaCl, 2% Triton X-100, 1 mM EDTA, 20 mM tris-HCl, pH 8.0) and sonicated as indicated above. Samples were then centrifuged (43,700× *g*, 4 °C, 60 min), the pellet was discarded, and the supernatant was transferred to cellulose dialysis tubes (14 kDa cut-off pore size). To gradually remove urea from the medium, four buffers with decreasing urea concentrations were prepared (1.5, 1, 0.5, and 0 M urea in 0.5 M NaCl, 20 mM tris-HCl, pH 8.0). Dialyses were carried out overnight at 4 °C under constant stirring for at least 4 h in each buffer to allow proper refolding of the protein [39]. Finally, EDTA (1 mM final concentration) was added to the protein solution, and urea-free 6His-GST-*Pf*DXRt-6His was purified by immobilized metal affinity chromatography (IMAC). Briefly, samples were applied onto 1-mL His-Trap FF columns (GE Healthcare, Chicago, IL, USA) equilibrated with buffer A (100 mM NaCl, 10 mM imidazole, 40 mM tris-HCl, pH 8.0) and washed with 25 column volumes of the same buffer. The recombinant enzyme was eluted with a gradient of imidazole from 10 to 500 mM prepared using buffer A and buffer B (100 mM NaCl, 500 mM imidazole, 40 mM tris-HCl, pH 8.0). The different fractions were analyzed by SDS-PAGE followed by Coomassie blue staining, and those containing 6His-GST-*Pf*DXRt-6His were subsequently combined and concentrated using Amicon Ultra-15 10 kDa cut-off centrifugal filters (Merck Millipore, Burlington, MA, USA) and stored at −20 °C in the presence of 50% glycerol until used. For the experiments carried out in this study, preparations were desalted using Zeba™ Spin desalting columns (Thermo Fisher Scientific, Waltham, MA, USA). Protein concentration in the samples was determined using the Pierce™ BCA Protein Assay Kit (Thermo Scientific, Rockford, IL, USA) following the manufacturer’s protocol.

To obtain a GST-free enzyme for aptamer selection (see below), the synthetic DNA sequence coding for *Pf*DXRt (described above) was cloned into the pQE30 plasmid vector (Qiagen, Hilden, Germany; Supplementary Figure S2), which incorporates a 6× His-tag at the N-terminus of the recombinant protein. The resulting pQE30-*Pf*DXRt construct was used to transform competent M15 *E. coli* cells (Qiagen), and purified 6His-*Pf*DXRt preparations were obtained following the IMAC protocol described above.

The *dxr* gene coding for *E. coli* DXR (*Ec*DXR, UniProt P45568) was synthesized (GenScript Biotech B.V.) and cloned into the pQE60 plasmid (GenScript Biotech B.V.; Supplementary Figure S3) used to express C-terminal 6× His-tagged proteins. Competent M15 *E. coli* cells were transformed with the resulting pQE60-*Ec*DXR construct, and purified preparations of *Ec*DXR-6His were obtained following the IMAC procedure described above.

### 2.2. In Vitro Selection of DNA Aptamers

**Figure 2:**
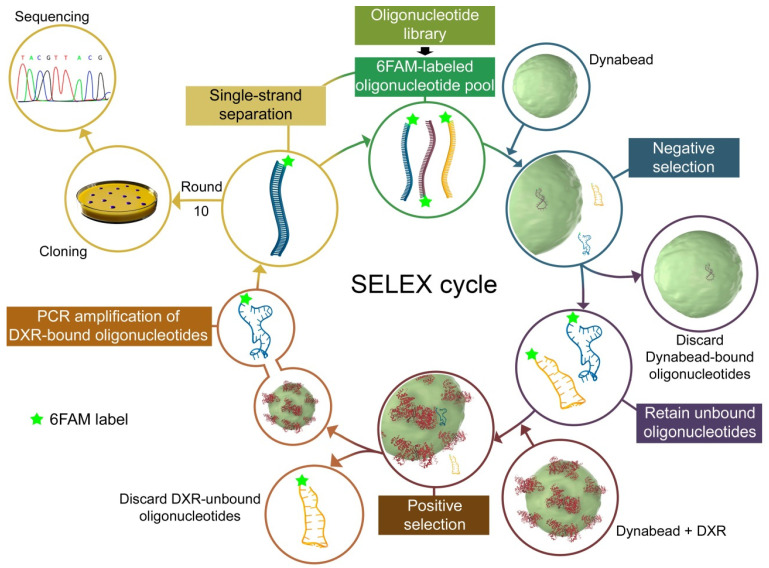
Scheme of the SELEX process used in this work to obtain DNA aptamers against *Pf*DXRt.

Aptamer candidates against 6His-*Pf*DXRt were selected using the Mag-SELEX method [35,36,40] (Figure 2). Briefly, 100 μL of 6His-*Pf*DXRt enzyme preparations (0.28 μg/μL) were incubated with 50 μL of cobalt magnetic beads (Dynabeads^®^, His-tag Isolation & Pulldown, Life Technologies, Carlsbad, CA, USA) pre-equilibrated in binding buffer (10 mM NaCl, 0.02% Tween-20, 50 mM monobasic sodium phosphate, pH 8.0). The Dynabead-6His-*Pf*DXRt complex was incubated at room temperature (RT) with 100 μL of a 10 ng/μL single-strand nucleic acid 76-mer library (up to 10^15^ sequences) with invariant PCR primer-binding flanking regions on each end and a randomized central sequence of 40 nucleotides (ATACCAGCTTATTCAATTN_40_AGATAGTAAGTGCAATCT, 1 µmol, DNA Technology A/S, Denmark). Prior to adding them to the enzyme-coated beads, oligonucleotides were allowed to fold into their native 3D conformations by a sequential incubation at 90, 5, and 37 °C (for 10, 15, and 8 min, respectively); this pre-folding step was done for every SELEX cycle and for all the binding, targeting, and activity assays reported in this work. Sequences that did not bind the 6His-*Pf*DXRt-functionalized beads were removed by hand pipetting the supernatant after capturing the beads using a magnetic separator (MagnetoPURE, Chemicell GmbH, Berlin, Germany), and the ssDNA bound to the Dynabead-6His-*Pf*DXRt complex was eluted in binding buffer at 80 °C for 5 min. In each SELEX cycle, a negative selection step was performed, incubating the aptamer pool with the protein-free beads to discard oligonucleotides with an affinity toward unmodified beads. After positive selection, the eluted sequences were amplified by PCR using *Pfu* DNA polymerase (Biotools B&M Labs, Madrid, Spain)*,* which is highly thermostable and shows proofreading activity [41]. As a rule, 20 cycles were programmed in a DNA 2720 Thermal Cycler (Applied Biosystems, Thermo Fisher Scientific): 94 °C, 60 s/46 °C, 30 s/72 °C, 30 s, with a 1-min 94 °C extra incubation before the first cycle. The 5′ ends of forward (5′-ATACCAGCTTATTCAATT-3′) and reverse (5′-AGATTGCACTTACTATCT-3′) primers were derivatized with 6-carboxyfluorescein (6FAM) and tri-biotin, respectively. The PCR products were purified using the NucleoSpin^TM^ Gel and PCR Clean-up kit (Macherey-Nagel GmbH & Co. KG, Düren, Germany) and loaded in a column (Micro Bio-Spin™ Chromatography Colums; Bio-Rad Laboratories, Inc., Hercules, CA, USA) containing NeutrAvidin^TM^ Agarose resin (Pierce, Thermo Fisher Scientific), from where the nonbiotinylated, fluorescein-labeled complementary strand was eluted with 0.1 M NaOH preheated to 96 °C. ssDNA was quantified using a NanoDrop 2000 spectrophotometer (Thermo Fisher Scientific) before entering the next cycle. This process was repeated for 10 such rounds of binding and selection until a pool of aptamers that bound fixed pRBCs with the desired specificity and affinity, as assessed by flow cytometry (see Section 2.10), was identified. Knowing the initial load of ssDNA entering a SELEX cycle (ssDNA_in_) and the amount of ssDNA eluted from the Dynabead-6His-*Pf*DXRt suspension at the end of each selection round (ssDNA_out_), the yield of binding ssDNA was calculated ([ssDNA_out_/ssDNA_in_] × 100).

### 2.3. Subcloning and Sequencing of Candidate Oligonucleotides

After 10 rounds of selection, the enriched oligonucleotide pool from SELEX cycles 8, 9, and 10 was PCR-amplified using unlabeled forward and reverse primers and *Pfu* DNA polymerase. The resulting products were cloned into the pBluescript SK+ plasmid after its linearization with *Sma*I (New England Biolabs, Ipswich, MA, USA) using T4 DNA ligase (New England Biolabs), and the ligation product was used for the heat-shock transformation of competent TOP10 *E. coli* cells (Life Technologies). The transformed cells were grown overnight at 37 °C in LB-agar plates containing 100 µg/mL ampicillin, and the recombinant colonies were differentiated with the blue/white screening method after the induction of *lacZ* expression in the presence of 5-bromo-4-chloro-3-indolyl-β-galactopyranoside (X-gal) and IPTG. White clones were randomly chosen from the plates, grown in 5 mL of LB (37 °C, 180 rpm, overnight), and their plasmids were isolated with the NucleoSpin plasmid kit (Macherey-Nagel GmbH & Co. KG) and quantified in a NanoDrop 2000 spectrophotometer. The successful insertion of sequences from the original library was validated by PCR with the specific forward and reverse primers using a GoTaq Green Master Mix (Promega Corporation, Madison, WI, USA)**.** DNA bands with the expected lengths were detected in 3% agarose gels stained with SyBR^®^ Safe DNA Gel Stain (Invitrogen, Thermo Fisher Scientific) and visualized under UV light. Ninety-six positive clones were sequenced using the M13 universal primer (Sanger sequencing service, GENEWIZ GmbH, Leipzig, Germany; https://www.genewiz.com/en-GB/Public/Services/Sanger-Sequencing, accessed on 10 January 2019). The selected sequences were chemically synthesized (Sigma-Aldrich Corporation) in sufficiently large amounts for the characterization of their binding to DXR.

### 2.4. Computational Analysis

Structures for *Pf*DXRt and *Ec*DXR were obtained from the protein data bank (PDB accession codes 5JMW and 1Q0Q, respectively). The aptamer structure was built from the sequence following the previously described workflow [42]. First, the mFold web server [43] built the ssDNA secondary structure from the initial sequence. Second, RNAComposer [44] was used to construct a refined 3D equivalent of RNA. Third, 3DNA [45] performed the translation from ssRNA to ssDNA, and finally, the refinement of the resulting structure (energetic minimization, addition of water) was done with YASARA [46,47]. All the protein-ssDNA dockings were performed with the HDOCK web server [48,49]. Figures were created using the 3D Protein Imaging server [50].

### 2.5. Western Blots

Proteins (20 µg) were fractionated in 12.5% SDS-PAGE gels (120 V, 45 min, RT) and electro-transferred for 1 h onto a preactivated polyvinylidene difluoride membrane at 4 °C and 400 mA. The membrane was washed (2×, 5 min) with phosphate-buffered saline, pH 7.4 (PBS), and blocked under orbital stirring (50 rpm) at RT for 1 h with 5% (*w/v*) skim milk powder in PBS containing 1% Triton X-100, washed again (3×, 5 min), and the aptamers labeled at their 5’-ends with 6FAM (λex/em: 495/520 nm) or with biotin, were added at a final concentration of 0.9 µM and incubated overnight. After washing (3×, PBS), 6FAM fluorescence was measured in an ImageQuant^™^ LAS 4000 CCD camera system (GE Healthcare) using epi-illumination and a Y515 filter. Biotin-labeled aptamers were revealed after incubation with horseradish peroxidase-conjugated streptavidin (Merck KGaA) and reacted with enhanced chemiluminescence substrate (Merck KGaA) prior to detection in an ImageQuant^™^ LAS 4000 CCD camera system.

### 2.6. Electrophoretic Mobility Shift Assay

Purified preparations of 6His-GST-*Pf*DXRt-6His, *Ec*DXR-6His, GST (G6511, Sigma-Aldrich Corporation), and bovine serum albumin (BSA) stored at −20 °C were desalted and equilibrated at ca. 0.1 mg/mL in 10 mM NaCl, 0.02% Tween-20, 50 mM HEPES, pH 8.0. After that, 14 µL of dilutions of each protein in the same buffer were mixed with 1 µL of chemically synthesized aptamer solution (150 ng) prepared in 0.5× TB buffer (45 mM tris-HCl, 45 mM boric acid, pH 8.0), which was previously allowed to pre-fold as described above, and incubated at RT for 60 min. Finally, samples were loaded into 2% agarose gels run in 0.5× TB at 20 V/cm, stained with SyBR^®^ Safe DNA Gel Stain, and imaged in an ImageQuant^™^ LAS 4000 CCD camera system.

### 2.7. Determination of Aptamer Dissociation Constants

The dissociation constants (*K_D_*) for the aptamers selected in this study were obtained by analyzing the binding of the fluorescently labeled ssDNA aptamer to target-functionalized beads. Briefly, 20 μg of *Ec*DXR-6His, 6His-GST-*Pf*DXRt-6His, GST, or BSA (the last two proteins as negative control) were incubated (15 min, RT, PBS) with 5 μL of 4% *w/v* 4-μm-diameter aldehyde/sulfate latex beads (Thermo Fisher Scientific) in a final volume of 20 μL. The mixture was then transferred to 1 mL PBS and incubated at 4 °C with gentle orbital stirring overnight. After centrifugation, the protein-coated beads were washed (3×, PBS), taken up in 50 μL PBS, and incubated (60 min, RT, in the dark) with 6FAM-labeled aptamers (previously allowed to fold as described above) in a series of dilutions (5.4, 2.7, 1.4, 0.7 and 0.3 µM) and analyzed by flow cytometry in a BD FACSAria II flow cytometer (BD Biosciences, Heidelberg, Germany) using a 488 nm laser line with a 530/30 BP filter and FACSDiva software supplied with the instrument. Equilibrium *K_D_* and the maximum number of binding sites (Bmax) of DXR-aptamer interactions were obtained by fitting the dependence of relative fluorescence intensity of specific binding on aptamer concentrations to the equation Y = B_max_ X/(K_D_ + X) [51]. GraphPad Prism 9 (GraphPad Software, San Diego, CA, USA) was used to plot the saturation curve, choosing nonlinear regression, the panel of saturation binding equations, and one site-specific binding. In addition, the Scatchard plot, which plots bound ligand vs. the ratio bound/free ligand, was also represented.

### 2.8. DXR Activity Assay

The *Ec*DXR-6His activity was monitored spectrophotometrically in an Ultrospec 3300 Pro spectrophotometer (GE Healthcare) at 340 nm by following oxidation of NADPH at RT in quartz cuvettes. Reactions were carried out in 500 µL of 100 mM tris-HCl buffer, pH 7.5, containing 1 mM MnCl_2,_ 0.15 mM NADPH (Ref. N5130, Sigma-Aldrich Corporation), 0.15 mM DXP (Ref. 13368, Sigma-Aldrich Corporation), 10 mM DTT, and 10 µg of *Ec*DXR-6His. Prior to its incorporation into the reaction, the aptamer was allowed to fold as described above. The GraphPad Prism 9 was used to calculate IC_50_ choosing the equation inhibitor concentration vs. normalized response.

### 2.9. P. falciparum Cultures

The *P. falciparum* 3D7 strain was grown in vitro in group B human erythrocytes using previously described conditions [52]. Parasites (thawed from glycerol stocks) were cultured at 37 °C in T-25 or T-175 flasks (Thermo Fisher Scientific) containing human erythrocytes at 3% hematocrit in Roswell Park Memorial Institute (RPMI) complete medium containing Albumax II (Gibco^TM^, Life Technologies), supplemented with 2 mM L-glutamine, under a gas mixture of 92.5% N_2_, 5.5% CO_2_, and 2% O_2_. Parasitemia was determined by microscopic counting of blood smears briefly fixed with methanol and stained for 10 min with Giemsa (Merck KGaA) diluted 1:10 in Sorenson’s buffer, pH 7.2.

### 2.10. Confocal Fluorescence Microscopy and Flow Cytometry Analysis

A 15-mL RBC culture at 3% hematocrit infected with the *P. falciparum* 3D7 strain at 3% parasitemia was washed with RPMI, fixed for 30 min at RT with 3% paraformaldehyde in PBS, and further washed with PBS (500× *g*, 5 min) until no hemolysis was observed. The cell pellet was then taken up in 50 µL of blocking medium (PBS supplemented with 5 mM MgCl_2_ and 1 mg/mL BSA), gently stirred for 5 min, and incubated at 37 °C for 1 h in the presence of 1.2 µM fluorescein-labeled aptamers. Prior to incubation with the cells, the aptamers (at 2.4 µM concentration) were allowed to fold into their native 3D conformations as described above. After rinsing with PBS, the cells were stained for 30 min at RT with 4 μg/mL of the DNA dye Hoechst 33342, rinsed with PBS, and placed in an 8-well chamber slide system (Lab-Tek^®^II, Thermo Fisher Scientific), and observed with an LSM 800 confocal fluorescence microscope (Zeiss, Jena, Germany) with a 100× oil-immersion objective (NA 1.4). The fluorochromes were detected at λex/em 400/480 (Hoechst 33342), 492/617 (6FAM), and 644/700 (Cy5.5 and Alexa Fluor 647). Colocalization analysis was performed with a rabbit polyclonal anti-ferredoxin-NADP reductase antibody (Abcam, Cambridge, UK) at 1:100 dilution, which was detected with an Alexa Fluor 647-labeled goat anti-rabbit IgG secondary antibody (Invitrogen, Thermo Fisher Scientific) at 1:200 dilution, and with the Cy5.5-labeled aptamer 2008s, developed against *P. falciparum* lactate dehydrogenase (*Pf*LDH) [53]. Image analysis was done with the Fiji software image processing package [54], and the Manders’ overlap coefficient calculation was determined using the JaCoP plug-in [55] for the Fiji software.

For flow cytometry analysis, fixed pRBCs were diluted in PBS to a final concentration of 1–10 × 10^6^ cells/mL, and samples were analyzed using an LSRFortessa^TM^ flow cytometer (BD Biosciences) set up with the 5 lasers, 20 parameters standard configuration. The single-cell population was selected on a forward-side scattergram. The fluorochromes were excited using 350 (Hoechst 33342), 488 (6FAM), and 640 nm lasers (Cy5.5 and Alexa Fluor 647), and their respective emissions were collected with 450/50, 530/30, 730/45, and 670/14 nm filters. The analysis was done with FACSDiva software (BD Biosciences) and Flowing Software 2.5.1 (www.btk.fi/cell-imaging; Cell Imaging Core, Turku Center for Biotechnology, Finland).

### 2.11. Bacterial Cultures

The bacterial strains *E*. *coli* ATCC 25,922 (American Type Culture Collection, Georgetown, DC, USA) and *Pseudomonas aeruginosa* PAO B+ isolate (kindly donated by Prof. Sara Soto, ISGlobal) were maintained in LB medium, whereas the *Enterococcus faecalis* Ef1 isolate (kindly donated by Prof. Sara Soto) was maintained in Tryptic Soy Broth. The concentration of cells was determined by serial dilution with subsequent plating on agar plates and measurement of colony-forming units (CFUs). To test the effect of aptamers on *E. coli*, a bacterial culture treated with 50 µM aptamer was exposed to heat shock as previously described [56]. For fluorescence microscopy studies, ca. 10^9^ CFUs were washed three times in PBS (10,000× *g*, 4 °C, 5 min), fixed with 4% paraformaldehyde (preheated at 60 °C) at RT for 1 h, and washed with PBS again. After that, the cells were immersed in 1 mL of blocking buffer (3% BSA in PBS) for 30 min at RT under 300 rpm shaking, washed two times, and then incubated at 4 °C overnight in the dark with 1.2 µM aptamers. Excess aptamers were washed away with PBS, and the fluorescence was observed in an 8-well Lab-Tek^®^II chamber slide system using a Zeiss LSM 800 confocal laser scanning microscope with a 100× oil-immersion objective (NA 1.4). 6FAM was detected at λex/em 492/617.

### 2.12. Ethics Statement

The human blood used in this work was from voluntary donors and commercially obtained from the *Banc de Sang i Teixits* (www.bancsang.net, accessed on 6 April 2020). Blood was not collected specifically for this research; the purchased units had been discarded for transfusion, usually because of an excess of blood relative to the anticoagulant solution. Prior to their use, blood units underwent the analytical checks specified in the current legislation. Before being delivered to us, unit data were anonymized and irreversibly dissociated, and any identification tags or labels were removed in order to guarantee the non-identification of the blood donor. No blood data were or will be supplied, in accordance with the current Spanish *Ley Orgánica de Protección de Datos* and *Ley de Investigación Biomédica*. The blood samples will not be used for studies other than those made explicit in this research.

## 3. Results and Discussion

### 3.1. Aptamer Selection

**Figure 3:**
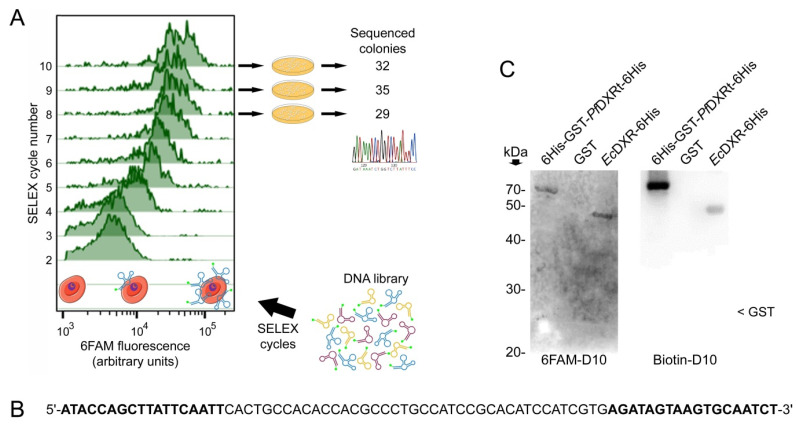
Selection of specific aptamers against *Pf*DXR. (**A**) Schematic rendering of the progressive selection of pRBC-binding 6FAM-labeled aptamers along the SELEX cycles 2 to 10. The enrichment in specific aptamers originally present in the randomly synthesized DNA library is reflected by an increase in the binding of the selected fluorescent oligonucleotides to pRBCs, which become permeable to aptamers after the fixation step prior to the flow cytometry analysis shown. (**B**) D10 sequence, showing in bold the PCR primer-binding regions. (**C**) Western blot analysis of D10 binding to 6His-GST-*Pf*DXRt-6His and *Ec*DXR-6His. The approximate electrophoretic mobility of free GST is indicated. Petri dish cartoon reproduced with permission (https://creativecommons.org/licenses/by-nc-nd/2.0/legalcode, accessed on 23 April 2021).

Flow cytometry analysis was used to follow after each SELEX cycle the enrichment in 6FAM-labeled aptamers binding pRBCs (Figure 3A), which, during the fixation process for flow cytometry, become permeable to the aptamers [34]. The SELEX cycles were halted at round 10 when the observed DXR-associated fluorescence was not significantly different from that detected in rounds 8 and 9. The oligonucleotide pool from rounds 8, 9, and 10 were subcloned in order to obtain plasmids containing individual aptamers. Ninety-six clones were sequenced (Table S1), and the most represented oligonucleotide (D10, Figure 3B), which was repeated 16 times, was chemically synthesized for further analysis. Since the yield of recombinant 6His-*Pf*DXRt was relatively low, a GST tag was incorporated to obtain the larger amounts of protein that would be required for subsequent characterization assays. In Western blots, 6FAM-labeled D10 bound both 6His-GST-*Pf*DXRt-6His and *Ec*DXR-6His with similar affinity (Figure 3C). The biotin-labeled aptamer, however, recognized 6His-GST-*Pf*DXRt-6His much more strongly than the *E. coli* enzyme. Since the SELEX selection was made with 6FAM-labeled oligonucleotides, the substitution of the fluorescent tag by biotin likely resulted in a slightly different 3D conformation which could affect target binding.

### 3.2. Characterization of Aptamer Binding to DXR In Vitro

**Figure 4:**
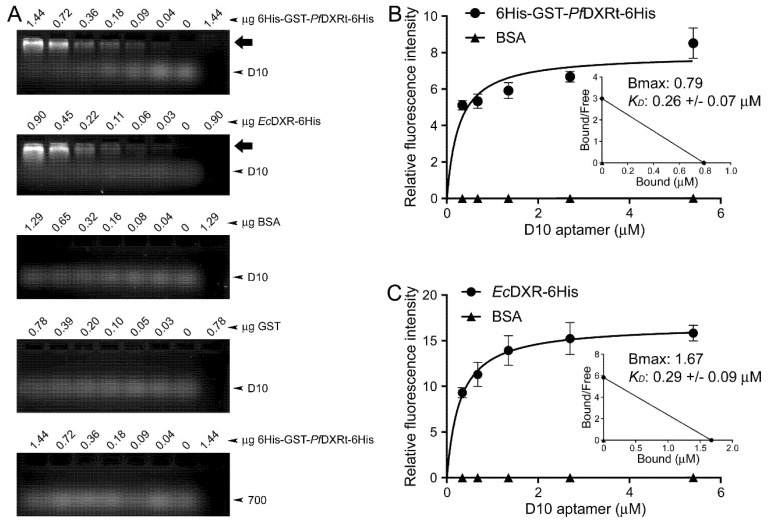
Characterization of the D10 aptamer evolved against *Pf*DXRt. (**A**) Electrophoretic mobility shift assay of the interaction of D10 with its target protein as part of the recombinant polypeptides 6His-GST-*Pf*DXRt-6His and *Ec*DXR-6His. The negative controls include analysis of D10 binding to BSA and GST and of aptamer 700 [34] to 6His-GST-*Pf*DXRt-6His. The arrows indicate the position of DXR-D10 complexes just entering the gel. The last lane on the right side of each gel contains no D10. (**B**,**C**) Determination of *K_D_* and Bmax for the binding of the 6FAM-labeled D10 aptamer to (**B**) 6His-GST-*Pf*DXRt-6His and (**C**) *Ec*DXR-6His. Insets: Scatchard plots. The negative control binding of D10 to free GST is presented in Figure S4.

To discard an effect of the fluorescein chemical group on the folding of the D10 conformation binding DXR, electrophoretic mobility shift assays were conducted to study the interaction of non-labeled D10 aptamer with 6His-GST-*Pf*DXRt-6His and *Ec*DXR-6His (Figure 4A). The retardation observed for D10 in agarose gels indicated its concentration-dependent interaction with DXR from both *P. falciparum* and *E. coli*. In-solution association analyses of the interaction between the 6FAM-labeled D10 aptamer and 6His-GST-*Pf*DXRt-6His indicated respective Bmax and *K_D_* values of 0.79 and 0.26 ± 0.07 µM (Figure 4B), which were similar to those obtained for the D10-*Ec*DXR-6His interaction (1.67 and 0.29 ± 0.09 µM, respectively; Figure 4C). The binding to GST was negligible (Supplementary Figure S4). These *K_D_* values were comparable to those of aptamers selected against *Pf*LDH [53] and glutamate dehydrogenase [57] (42 nM and 0.5 µM, respectively).

**Figure 5:**
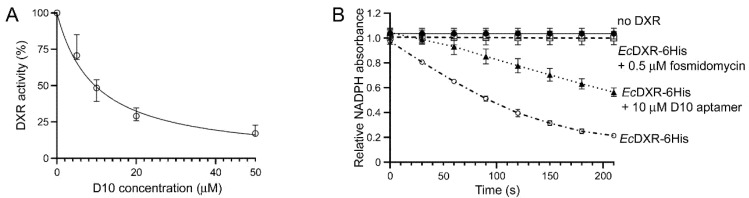
*Ec*DXR-6His in vitro activity assays. (**A**) DXR activity assays done at different D10 concentrations. (**B**) Positive (in the presence of DXR without inhibitor) and negative control activity assays (in the absence of DXR and in the presence of DXR with 0.5 µM fosmidomycin) compared to the test sample containing DXR and 10 µM D10.

Due to the low metabolic activity of the recombinant forms of *Pf*DXRt obtained for this work, in vitro DXR activity assays were conducted with the *E. coli* enzyme. The label-free D10 aptamer showed a modest concentration-dependent inhibitory activity of *Ec*DXR-6His (Figure 5A), with an IC_50_ of 9.6 µM (8.7 µM/10.6 µM confidence interval, 95% confidence level) that, nevertheless, was significantly weaker than that obtained for fosmidomycin (Figure 5B), whose reported IC_50_ for recombinant *Pf*DXR is 32 nM [58]. This result suggested that D10 binds the enzyme in a position that does not completely block the active center, in agreement with the small areas recognized by aptamers in target molecules [31,36,59,60,61,62]. Accordingly, no inhibitory activity was observed in *E. coli* cultures up to a D10 concentration of 50 µM (Figure S5).

The electrophoretic mobility shift analysis data and association assays in solution indicated that DXR is bound by unmodified D10 (Figure 4) but also by the aptamer functionalized with 6FAM or biotin, according to Western blot data (Figure 3). Other studies have shown that aptamers raised against *Pf*LDH can be incorporated into large structures, such as DNA origami, without losing their ability to bind the target protein [62]. The *Pf*LDH captured by aptamer-modified DNA origami retained its enzymatic activity, in agreement with our results, indicating a good binding of D10 to DXR, which, however, at 10 µM does not completely inhibit the *E. coli* enzyme’s capacity to metabolize its substrate.

**Figure 6:**
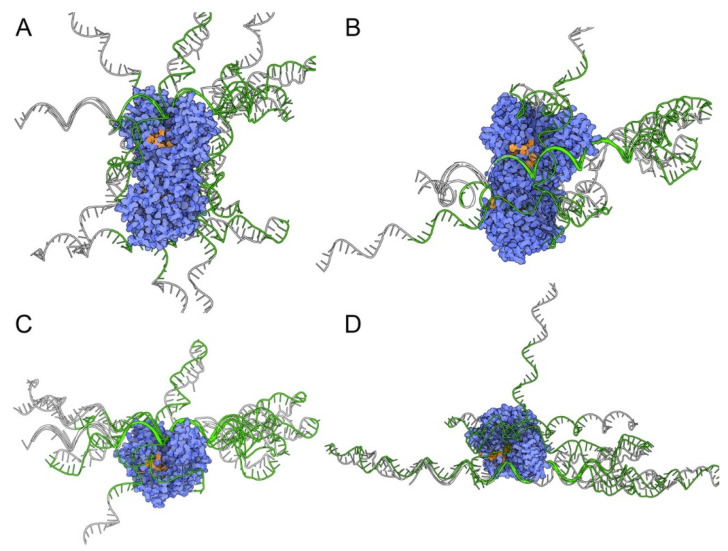
Computational simulations to depict the most favorable D10-DXR interactions. Top 10 energetically favorable D10 (colored green) docking for DXR (colored blue) for the homodimeric active enzyme found in the cell of (**A**) *Ec*DXR (PDB code 1Q0Q) and (**B**) *Pf*DXRt (PDB code 5JMW) and their respective monomeric forms (**C**,**D**), which were used in the SELEX cycles. The enzyme active site cavity is colored orange. Data for delimiting the active site was obtained from [63,64,65,66].

Because the full DXRt sequence was used in the SELEX process, the likelihood that the selected aptamers bound the active site of the enzyme was low, given the large size of the protein (47.3 kDa for the mature form) relative to the small dimensions of the antigens recognized by the aptamers [36,61]. Bioinformatic structure-based analysis suggested that the most favorable D10-DXR interactions were restricted to the solvent-exposed sides of the proteins (Figure 6, Table S2 and Supplementary Videos S1–S4). Consistent with the observed low level of suppression of enzymatic activity by D10, the predicted interactions with active center amino acid residues were 4.7% for *Pf*DXRt (mainly the exposed regions of W296 and P358) and 0.2% for *Ec*DXR (M214). Notably, the vast majority of predicted interactions with *Ec*DXR and *Pf*DXRt (83.2% and 88.4%, respectively) were mediated by the selected 40-nucleotide central region of D10 (C19-G58).

Future antimalarial therapeutic aptamers inhibiting (in the nM range) this essential *Plasmodium* enzyme could be obtained through epitopic targeting, an experimental approach that has already shown its potential for the development of aptamers against discrete *Pf*LDH regions [60]. Most *Plasmodium*-specific aptamers have as proposed applications diagnosis, drug delivery, the study of molecular mechanisms, and protein purification [29], and just a handful of works describe aptamers for therapeutic applications, targeting adhesion proteins of the parasite such as EMP1 [31] and Var2CSA [67] or the heme group released upon hemoglobin digestion [32]. An aptamer inhibiting the function of the *Leishmania infantum* poly-A binding protein has potential for future therapies against leishmaniasis [68]. However, no antimalarial therapeutic aptamer whose activity relies on enzymatic inhibition has been developed so far. Epitopic targeting of the active center of DXR and other MEP pathway enzymes could open a wide new avenue of aptamer-based antimalarial compounds. Alternatively, D10 could be modified with chemical moieties having DXR inhibitory activity, such as fosmidomycin analogs or yet-to-be-discovered molecules that might benefit from D10 targeting to DXR in order to increase their antimalarial activities. As an example of this strategy, an aptamer-ampicillin conjugate was constructed to impart an improved biofilm penetration and bacteria-killing efficacy to the antibiotic [69].

### 3.3. Characterization of Aptamer Binding to Plasmodium Cells

**Figure 7:**
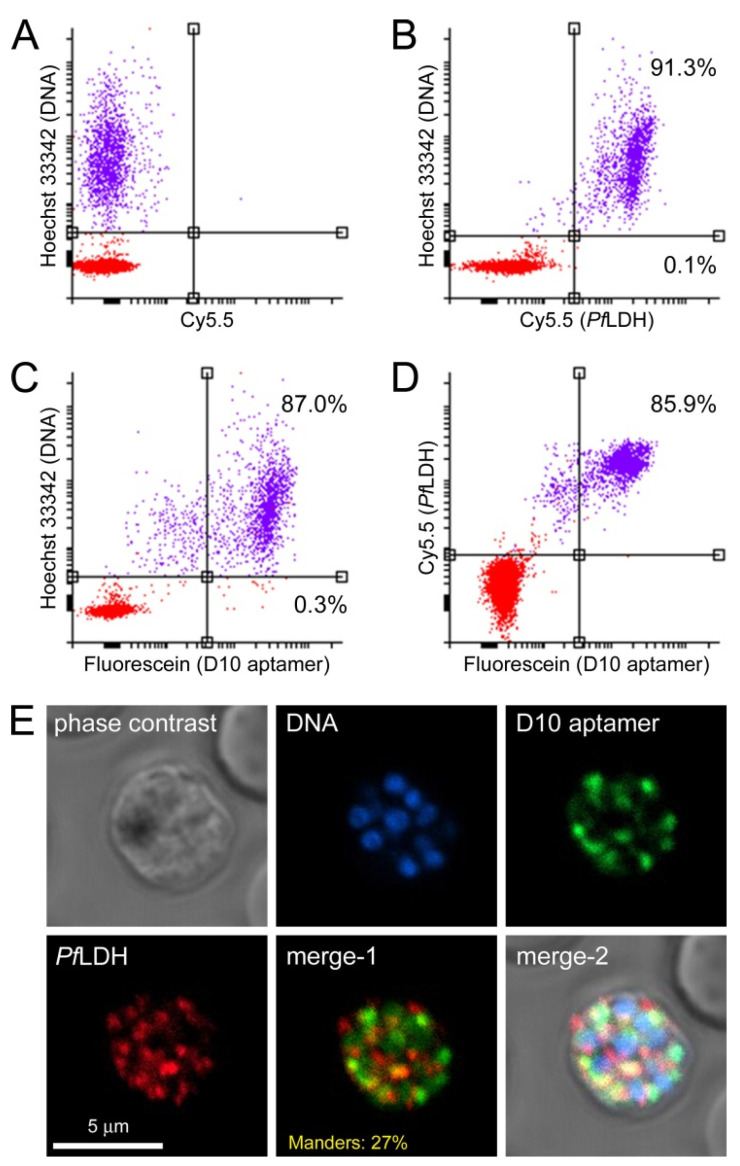
Colocalization study in *P. falciparum* of D10 and *Pf*LDH detected with the 2008s aptamer. (**A**–**D**) Flow cytometry analysis of (**B**) *Pf*LDH, (**C**) D10, and (**D**) both molecules together. The axis scales indicate relative fluorescence arbitrary units. Panel (**A**) shows a control with Hoechst 33342 DNA staining only. Percentages in panels (**B**,**C**) indicate the fraction of labeled cells relative to the total number of (**upper panels**) pRBCs and (**lower panels**) non-infected RBCs. The percentage in panel (**D**) indicates the fraction of cells positive for D10 and *Pf*LDH relative to the total cells positive for D10 or *Pf*LDH. (**E**) Confocal fluorescence microscopy analysis (merge-1: red and green channels; merge-2: all channels). The Manders’ overlap coefficient is indicated in the **merge-1 panel**.

The low activity of D10 as a DXR inhibitor led us to explore if it could be used as an apicoplast marker for eventual applications in cell biology research and malaria diagnosis. In colocalization studies done in *P. falciparum* cultures with the 2008s aptamer against the cytosolic enzyme *Pf*LDH [53], 6FAM-labeled D10 specifically discriminated non-parasitized red blood cells from *Plasmodium*-infected RBCs, according to flow cytometry (Figure 7A–D). The confocal fluorescence microscopy data of the subcellular targeting of 6FAM-labeled D10 in pRBCs showed a punctated pattern consistent with an apicoplastidic localization (Figure 7E and Supplementary Video S5). The low Manders’ overlap correlation coefficient (27%) is consistent with a preferentially non-cytosolic localization of D10.

**Figure 8:**
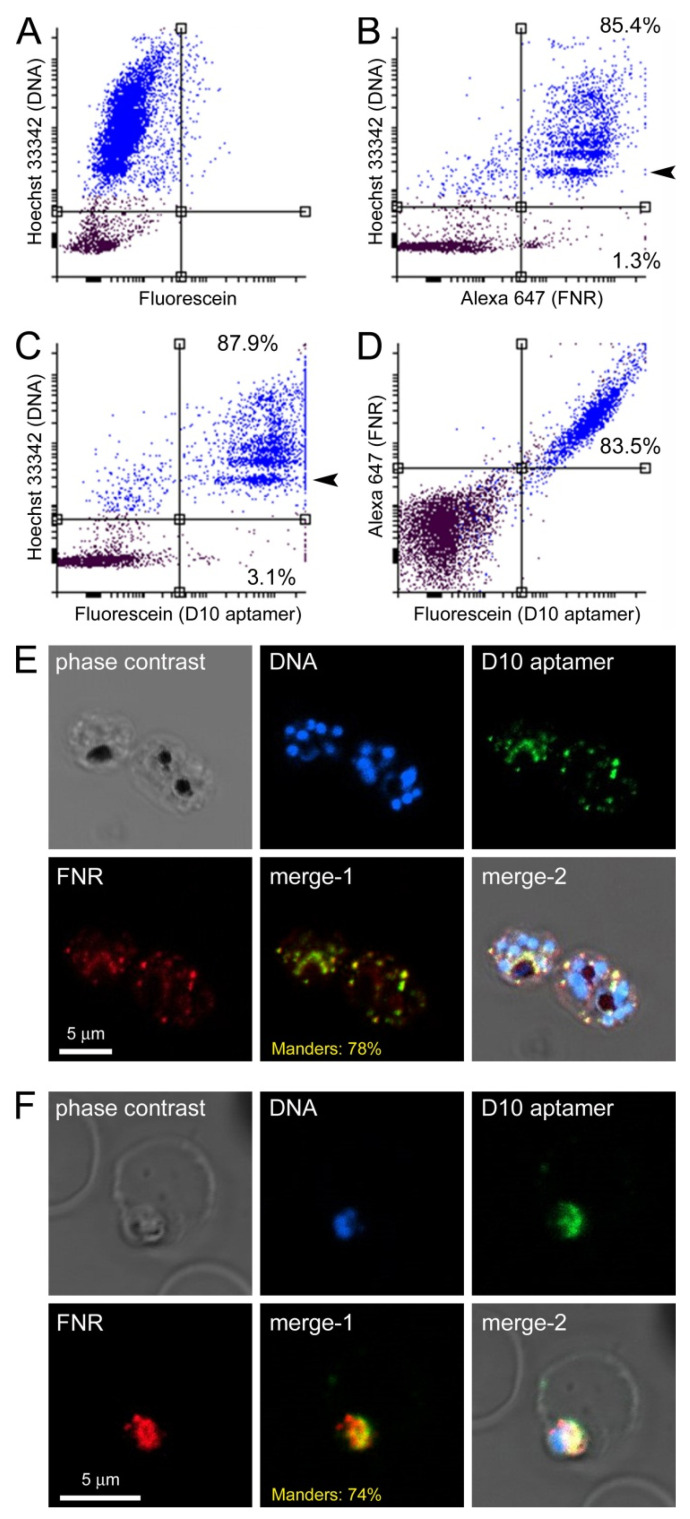
Colocalization study in *P. falciparum* of 6FAM-labeled D10 and FNR. (**A**–**D**) Flow cytometry analysis of (**B**) FNR, (**C**) D10, and (**D**) both molecules together. The axis scales indicate relative fluorescence arbitrary units. Panel (**A**) shows a control with Hoechst 33342 DNA staining only. The arrowheads in panels (**B**,**C**) indicate the ring stage population. Percentages in panels B and C indicate the fraction of labeled cells relative to the total number of (**upper panels**) pRBCs and (**lower panels**) non-infected RBCs. The percentage in panel (**D**) indicates the fraction of cells positive for D10 and FNR relative to the total of cells positive for D10 or FNR. (**E**,**F**) Confocal fluorescence microscopy analysis in (**E**) schizont and (**F**) ring stages (merge-1: red and green channels; merge-2: all channels). The Manders’ overlap correlation coefficients are indicated in the **merge-1 panels**.

D10 was observed to bind all the blood stages of the parasite, including ring forms (Figure 8C,F), in agreement with targeting the apicoplast, as this organelle is present in all the intraerythrocytic forms of the pathogen. Targeting the apicoplast was confirmed by fluorescence confocal microscopy (Figure 8E,F) through colocalization analysis with an antibody against the apicoplastidic enzyme ferredoxin-NADP reductase (FNR).

**Figure 9:**
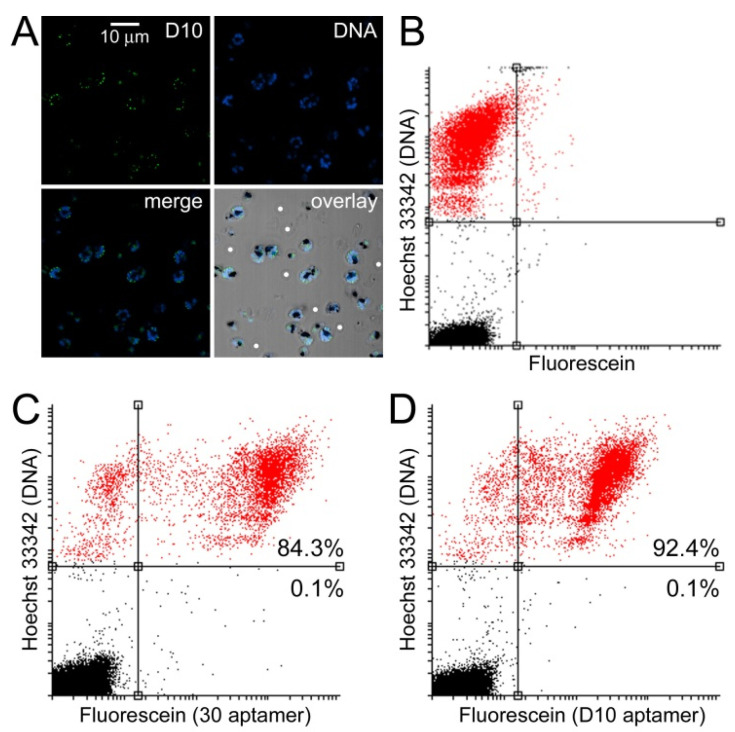
Comparison of the targeting to pRBCs of D10 and a previously described pRBC-specific aptamer. (**A**) Fluorescence confocal microscopy analysis of the specific targeting to pRBCs of 6FAM-labeled aptamer D10. White dots indicate some non-infected RBCs. (**B**–**D**) Flow cytometry analysis. (**B**) Hoechst 33342-only control. (**C**) Targeting analysis of 6FAM-labeled aptamer 30. (**D**) Targeting analysis of 6FAM-labeled aptamer D10. Percentages indicate the fraction of labeled cells relative to the total number of (**upper panels**) pRBCs and (**lower panels**) non-infected RBCs.

In previous work, we presented the selection of a family of DNA aptamers exhibiting highly specific discrimination of RBCs vs. pRBCs [34]. When comparing D10 with aptamer 30, one of the sequences described in the article mentioned above, a similar binding specificity was observed for both aptamers (Figure 9), which suggested that D10 can be a valuable element for future malaria diagnostic approaches.

### 3.4. Characterization of Aptamer Binding to Bacterial Cells

**Figure 10:**
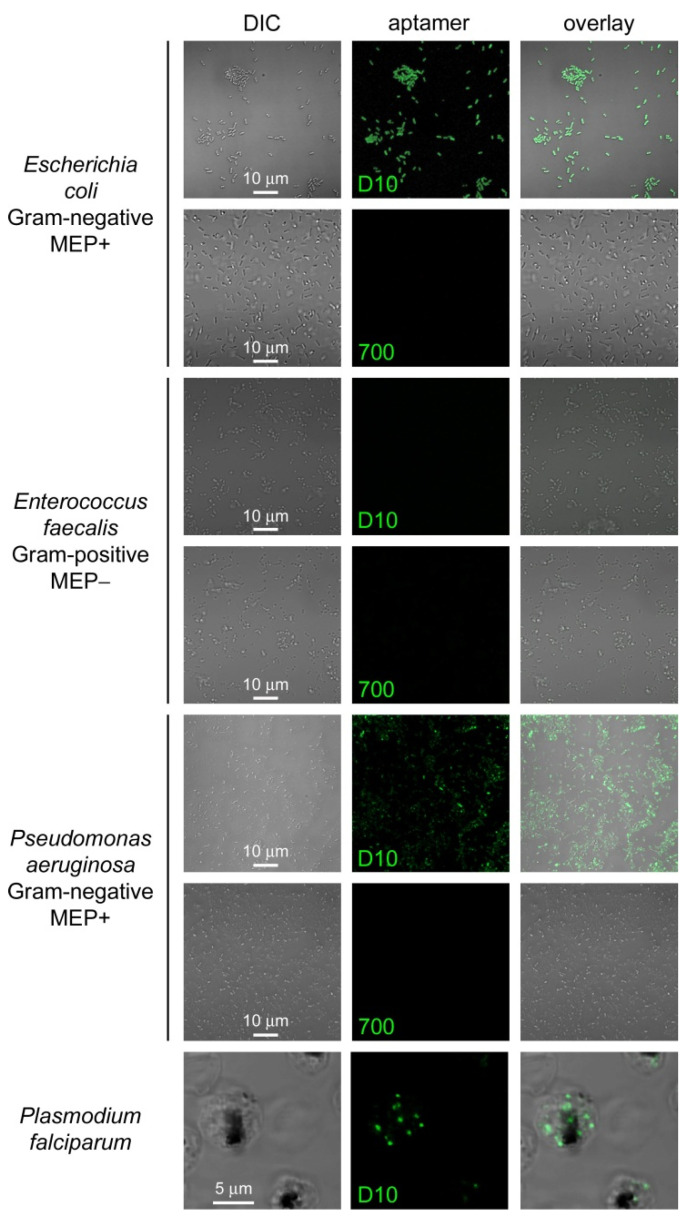
Fluorescence microscopy analysis of *E. coli*, *E. faecalis*, and *P. aeruginosa* cells treated with 1.2 µM 6FAM-labeled D10 aptamer. Positive and negative controls include the staining of *P. falciparum* with the same aptamer and the three bacteria species with an identical concentration of the 6FAM-labeled aptamer 700, respectively. DIC: differential interference contrast image.

Since the D10 aptamer bound and slightly inhibited *E. coli* DXR in vitro, we explored if it would also detect the enzyme in *E. coli* cell cultures in order to explore its potential for bacterial presence determination. Bacterial contamination has become an increasing concern in disease control, clinical diagnosis, environmental monitoring, and fouling analysis of foods, hospital settings, and surgical instrumentation [70]. Indeed, *E. coli* cells were strongly stained by 1.2 µM D10 but not by an identical concentration of the control aptamer 700 [34], of an equal length but with a random sequence not selected to bind DXR (Figure 10). D10 was also selective for the detection of other MEP+ bacteria (e.g., *P. aeruginosa*) vs. those lacking DXR (e.g., *E. faecalis*).

The MEP pathway is present in many human pathogenic bacteria, both gram-positive and gram-negative, such as *Bacillus anthracis*, *Clostridium* spp., *Listeria monocytogenes*, *Chlamydia* spp., *Salmonella enterica*, *Vibrio cholerae*, *Shigella* spp., *Neisseria* spp., or *Yersinia enterocolitica*, to name just a few [71]. The incorporation into biosensors of DNA aptamers such as D10, specifically targeting MEP pathway enzymes, could represent an important advance in the development of methods for the detection of pathogenic bacteria contamination. The immense versatility of DNA also offers perspectives for multiplexed detection devices where specific aptamers designed to identify individual pathogens can significantly improve current diagnostic methods.

The selection of D10 was made through a Mag-SELEX process where the target protein was immobilized and exposed to a sequentially and structurally diverse library of up to 10^15^ oligonucleotides. Our strategy based on subcloning followed by Sanger sequencing obtained an aptamer with excellent targeting specificity for *Pf*DXR in live parasite cells. However, next-generation sequencing of the oligonucleotide pool at the end of the SELEX cycles would have offered a greater number of sequences, thus allowing a more robust result and possibly the identification of potential aptamers with higher enzymatic inhibition capacities. Although SELEX has provided a toolbox of aptamers for multiple purposes, recent advances in bioinformatics resources now offer time- and cost-efficient alternatives for the development of novel oligonucleotides [42,43,44,45,46,47,48,49,72].

The vast majority of malaria rapid diagnostic tests (RDTs) manufactured, purchased, and used are based on the detection of *P. falciparum* histidine-rich protein 2 (*Pf*HRP2), alone or in combination with other antigens such as LDH and *Plasmodium* aldolase. These approaches based on antibodies still have some limitations [73]: they have short expiration times of around 9 months, their stability is compromised by high temperature and humidity in field settings, and they rely on animal or hybridoma production, which can be expensive and undergo batch production variability. Due to the intracellular nature of the parasite, there are few validated diagnosis targets, which are at risk of being lost in certain strains, as it has already been observed for *Pf*HRP2-based RDTs [74,75]. In addition, current RDTs are mostly developed for *P. falciparum*, and those targeting other species usually have less sensitivity [76]. Beyond their use as a diagnostic tool, the detection of *Plasmodium* antigens in blood samples is also employed to verify the elimination of the parasite after treatment, but the decay of parasite antigens takes longer than the clearance of parasitemia, leading to false positives [77]. Therefore, accurate detection of the disease is essential. Several nanotechnological tools have been assessed to strengthen the selection and application of aptamers for malaria diagnosis targeting *Pf*LDH, such as 2008s [53], pL1 [78,79], P38 [80], and LDHp 11 [60]. The 2008s ssDNA aptamer, with a high affinity for *Pf*LDH, has been incorporated into a colorimetric assay for the rapid diagnosis of malaria in blood samples [53,81], taking advantage of sequence and structural differences between the *Plasmodium* and human enzymes. Multi-target technologies such as aptamer-tethered enzyme capture [33] can help to determine if D10 will also have an affinity for DXR in other *Plasmodium* species, which will inform its potential as either a pan-malaria diagnosis tool or as a *P. falciparum*-specific marker. For instance, the need to discriminate between the two main human malaria causative parasites, *P. falciparum,* and *P. vivax*, should help to personalize treatments, possibly delaying resistance emergence and avoiding vivax malaria relapses [82]. Advances in this field have made use of the different affinities that aptamers such as 2008s, pL1, and LDHp11 show toward *P. vivax* and *P. falciparum* by integrating their electric sensor outputs in simple logical gates [83]. The accuracy of such promising approaches will benefit from the development of aptamers such as D10 that bind novel diagnostic targets in the pathogen.

For malaria research, the study of *Plasmodium* organelles and dynamic processes and changes in the metabolism of the parasite is of paramount importance. Visualization of the pathogen and its subcellular compartments greatly contributes to understanding its biology [84]. Few apicoplast markers are currently available, and to the best of our knowledge, all of them rely on the use of antibodies, e.g., against FNR [85,86] and the acyl carrier protein [87,88] or of apicoplast-targeted green fluorescent protein [89]. In this regard, the apicoplast-specific D10 aptamer tagged with fluorescent molecules or biotin represents an important addition to the small toolkit for the investigations of the cellular biology of malaria parasites.

Finally, DNA aptamers have been proposed as good candidates for bacteria sensing [70,90,91,92,93], which places aptamers such as D10, targeting MEP pathway enzymes, as interesting potential elements for the development of future biosensors of bacterial infections.

## 4. Conclusions

We have characterized D10, the first DNA aptamer targeting *P. falciparum* 1-deoxy-D-xylulose-5-phosphate reductoisomerase. D10 also binds the homologous *E. coli* enzyme, weakly inhibiting its in vitro activity. D10 is a specific marker of *Plasmodium*-infected erythrocytes and of the apicoplast organelle, which presents this aptamer as a potential element of future malaria diagnostic strategies and as a valuable tool for cellular biology studies in *Plasmodium* and other Apicomplexa. D10 also detects bacteria possessing the methyl erythritol phosphate pathway, a property that can be applied to the detection of certain microbial contaminations.

## Data Availability

All the data supporting the reported results can be found in the main article and in the Supplementary Materials files.

## Supplementary Materials

The following are available online at https://www.mdpi.com/article/10.3390/pharmaceutics14112515/s1, Figure S1: PfDXRt cloned into the pGS21a expression vector. Figure S2: PfDXRt cloned into the pQE30 expression vector. Figure S3: EcDXR cloned into the pQE60 expression vector. Figure S4: Negative control binding of D10 to free GST. Figure S5: Effect of 50 µM D10 on the growth of an E. coli culture. Table S1: Sequences of 96 subcloned individual oligonucleotides. Table S2: Top 10 energetically favorable protein-D10 interaction models according to HDOCK. Video S1–S4: Enzyme-aptamer interactions. Video S1: E. coli monomer; Video S2: E. coli dimer; Video S3: P. falciparum monomer; Video S4: P. falciparum dimer; Video S5: Confocal fluorescence microscopy analysis of D10 subcellular targeting in P. falciparum.
