# Supplementary material for: Selection of an Aptamer against the Enzyme 1-deoxy-D-xylulose-5-phosphate Reductoisomerase from Plasmodium falciparum
Source: Pharmaceutics. 2022 Nov 19;14(11):2515. doi: 10.3390/pharmaceutics14112515 (PMC9695703; doi:10.3390/pharmaceutics14112515)
Supplement: Supplementary file 1 [file pharmaceutics-14-02515-s001.zip › Supplementary Figures and Table S1_220831.pdf]

Supplementary Materials

Selection of an aptamer against the enzyme 1-deoxy-D-xylulose-5-phosphate reductoisomerase from *Plasmodium falciparum*

Carlota Roca, Yunuén Ávalos-Padilla, Beatriz Prieto-Simón, Valentín Iglesias, Miriam Ramírez, Santiago Imperial, Xavier Fernàndez-Busquets

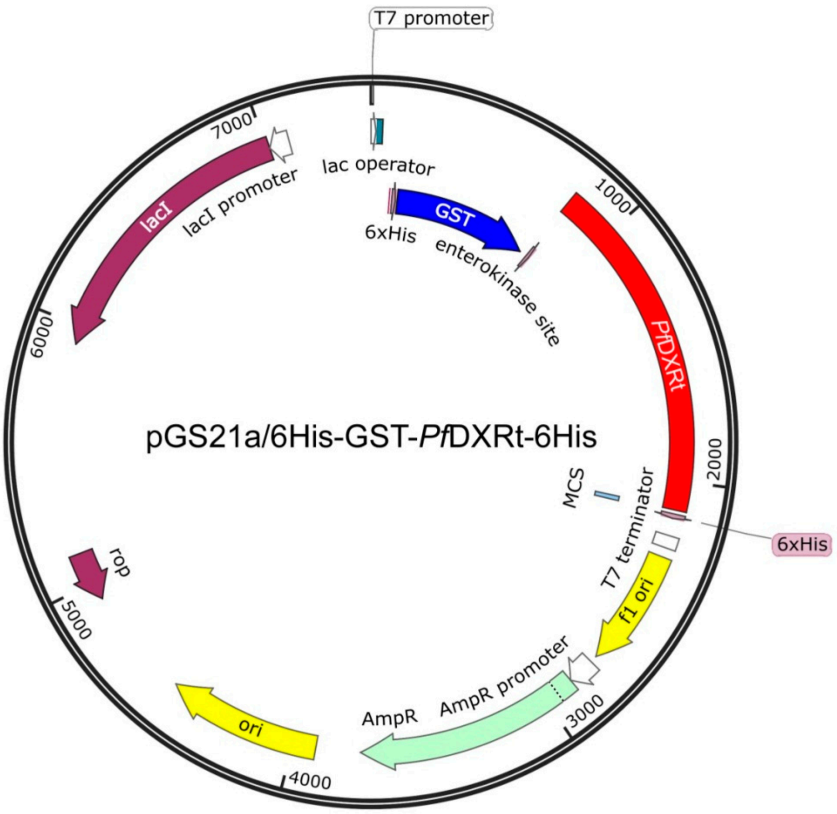

ATG-His tag-GST-enterokinase site-*Pf*DXRt-His tag-stop codon

Recombinant protein sequence (670 amino acids, 76,774 kDa):

**MSGSHHHHHHSSGMSPILGYWKIKGLVQPTRLLLEYLEEKYEEHLYERDEGDKWRNKKFELGLEFPNLPYYIDGDVKLTQSMAIIRYIA
DKHNMLGGCPKERAEISMLEGAVLDIRYGVSRIAYSKDFETLKVDFLSKLPEMLKMFEDRLCHKTYLNGDHVTHPDFMLYDALDVVLY
MDPMCLDAFPKLVCFKKRIEAIPQIDKYLKSSKYIAWPLQGWQATFGGGDHPPKSDLGHTGHRSGTDDDDKAIKKPINVAIFGSTGSIG
TNALNIIRECNKIENVFNVKALYVNKSVNELYEQAREFLPEYLCIHDKSVYEELKELVKNIKDYKPIILCGDEGMKEICSSNSIDKIVIGIDSF
QGLYSTMYAIMNNKIVALANKESIVSAGFFLKKLLNIHKNAKIIPVDSEHSAIFQCLDNNKVLKTKCLQDNFSKINNINKIFLCSSGGPFQN
LTMDELKNVTSENALKHPKWKMGKKITIDSATMMNKGLEVIETHFLFDVDYNDIEVIVHKECIIHSCVEFIDKSVISQMYYPDMQIPILYSLT
WPDRIKTNLKPLDLAQVSTLTFHKPSLEHFPCIKLAYQAGIKGNFYPTVLNASNEIANNLFLNNKIKYFDISSIISQVLESFNSQKVSENSE
DLMKQILQIHSWAKDKATDIYNKHNSSHHHHHH**

GST-*Pf*DXRt coding sequence (2,010 bases + stop codons):

**ATGTCTGGTTCTCATCATCATCATCATCATAGCAGCGGTATGTCCCCTATACTAGGTTATTGGAAAATTAAGGGCCTTGTGCAACC
CACTCGACTTCTTTTGGAATATCTTGAAGAAAAATATGAAGAGCATTTGTATGAGCGCGATGAAGGTGATAAATGGCGAAACAAA
AAGTTTGAATTGGGTTTGGAGTTTCCCAATCTTCCTTATTATATTGATGGTGATGTTAAATTAACACAGTCTATGGCCATCATACGT
TATATAGCTGACAAGCACAACATGTTGGGTGGTTGTCCAAAAGAGCGTGCAGAGATTTCAATGCTTGAAGGAGCGGTTTTGGATA
TTAGATACGGTGTTTCGAGAATTGCATATAGTAAAGACTTTGAAACTCTCAAAGTTGATTTTCTTAGCAAGCTACCTGAAATGCTG
AAAATGTTCGAAGATCGTTTATGTCATAAAACATATTTAAATGGTGATCATGTAACCCATCCTGACTTCATGTTGTATGACGCTCTT
GATGTTGTTTTATACATGGACCCAATGTGCCTGGATGCGTTCCCAAAATTAGTTTGTTTTAAAAAACGTATTGAAGCTATCCCACA
AATTGATAAGTACTTGAAATCCAGCAAGTATATAGCATGGCCTTTGCAGGGCTGGCAAGCCACGTTTGGTGGTGGCGACCATCCT
CCAAAATCGGATCTGGGCCACACAGGCCATAGATCTGGTACCGATGACGACGACAAGGCGATCAAGAAACCGATTAACGTGGC
GATTTTTGGCAGCACCGGCAGCATTGGCACCAACGCGCTGAACATTATCCGTGAGTGCAACAAGATCGAAAACGTGTTCAACGT
TAAAGCGCTGTACGTGAACAAGAGCGTTAACGAGCTGTATGAACAGGCGCGTGAGTTTCTGCCGGAATACCTGTGCATCCACGA
CAAAAGCGTGTATGAGGAACTGAAGGAGCTGGTTAAGAACATTAAAGACTACAAGCCGATCATTCTGTGCGGTGATGAGGGCAT
GAAAGAAATCTGCAGCAGCAACAGCATTGACAAGATCGTGATTGGTATCGATAGCTTCCAAGGCCTGTACAGCACCATGTATGC
GATCATGAACAACAAAATTGTGGCGCTGGCGAACAAGGAGAGCATCGTTAGCGCGGGTTTCTTTCTGAAGAAACTGCTGAACAT
TCACAAAAACGCGAAGATCATTCCGGTGGACAGCGAACACAGCGCGATCTTCCAGTGCCTGGATAACAACAAAGTTCTGAAGAC
CAAATGCCTGCAAGACAACTTCAGCAAGATCAACAACATCAACAAGATTTTCCTGTGCAGCAGCGGTGGCCCGTTTCAGAACCT
GACCATGGATGAGCTGAAAAACGTTACCAGCGAAAACGCGCTGAAGCACCCGAAGTGGAAAATGGGTAAGAAAATTACCATCG
ACAGCGCGACCATGATGAACAAGGGTCTGGAAGTGATCGAAACCCACTTCCTGTTTGACGTTGATTACAACGATATCGAAGTGA
TTGTTCACAAAGAATGCATCATTCACAGCTGCGTGGAGTTTATCGACAAGAGCGTTATTAGCCAGATGTACTATCCGGATATGCA
AATTCCGATCCTGTATAGCCTGACCTGGCCGGACCGTATCAAAACCAACCTGAAGCCGCTGGATCTGGCGCAGGTGAGCACCCT
GACCTTCCACAAGCCGAGCCTGGAACACTTTCCGTGCATCAAACTGGCGTACCAAGCGGGTATTAAGGGCAACTTCTATCCGAC
CGTTCTGAACGCGAGCAACGAGATCGCGAACAACCTGTTCCTGAACAACAAGATCAAGTACTTCGACATCAGCAGCATCATCAG
CCAGGTGCTGGAGAGCTTCAACAGCCAAAAAGTTAGCGAGAACAGCGAAGATCTGATGAAGCAGATCCTGCAAATTCACAGCT
GGGCGAAGGATAAAGCGACCGACATCTACAATAAGCACAATAGCAGCCACCACCACCACCACCACTAATGA**

Figure S1. *Pf*DXRt cloned into the pGS21a expression vector. Plasmid map, recombinant protein scheme and amino acid sequence, and DNA sequence of *Pf*DXRt.

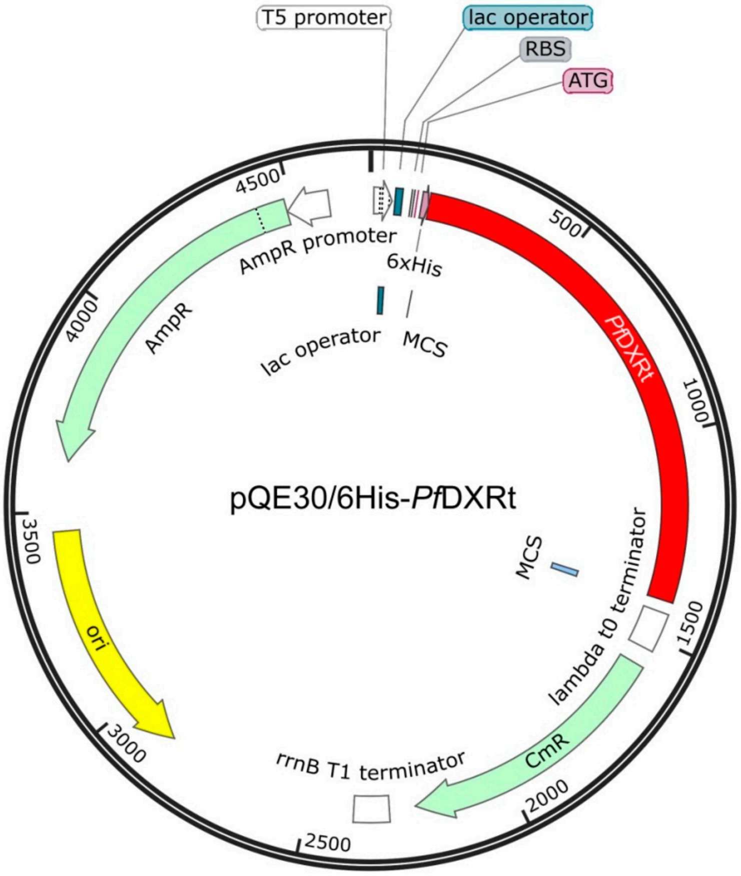

ATG-His tag-*Pf*DXRt-stop codon

Recombinant protein sequence (428 amino acids, 48,658 kDa):

**MRGSHHHHHHGSAIKKPINVAIFGSTGSIGTNALNIIRECNKIENVFNVKALYVNKSVNELYEQAREFLPEYLCIHDKSVYEELKELVKNIK
DYKPIILCGDEGMKEICSSNSIDKIVIGIDSFQGLYSTMYAIMNNKIVALANKESIVSAGFFLKKLLNIHKNAKIIPVDSEHSAIFQCLDNNKV
LKTKCLQDNFSKINNINKIFLCSSGGPFQNLTMDELKNVTSENALKHPKWKMGKKITIDSATMMNKGLEVIETHFLFDVDYNDIEVIVHKE
CIIHSCVEFIDKSVISQMYYPDMQIPILYSLTWPDRIKTNLKPLDLAQVSTLTFHKPSLEHFPCIKLAYQAGIKGNFYPTVLNASNEIANNLF
LNNKIKYFDISSIISQVLESFNSQKVSENSEDLMKQILQIHSWAKDKATDIYNKHNSS**

*Pf*DXRt coding sequence (1,284 bases + stop codon):

**ATGAGAGGATCGCATCACCATCACCATCACGGATCCGCGATTAAAAAACCGATTAACGTGGCGATTTTTGGCAGCACCGGCAGC
ATTGGCACCAACGCGCTGAACATTATTCGTGAATGCAACAAAATTGAAAACGTGTTTAACGTGAAAGCGCTGTATGTGAACAAAA
GCGTGAACGAACTGTATGAACAGGCGCGTGAATTTCTGCCGGAATATCTGTGCATTCATGATAAAAGCGTGTATGAAGAACTGA
AAGAACTGGTGAAAAACATTAAAGATTATAAACCGATTATTCTGTGCGGCGATGAAGGCATGAAAGAAATTTGCAGCAGCAACA
GCATTGATAAAATTGTGATTGGCATTGATAGCTTTCAGGGCCTGTATAGCACCATGTATGCGATTATGAACAACAAAATTGTGGC
GCTGGCGAACAAAGAAAGCATTGTGAGCGCGGGCTTTTTTCTGAAAAAACTGCTGAACATTCATAAAAACGCGAAAATTATTCCG
GTGGATAGCGAACATAGCGCGATTTTTCAGTGCCTGGATAACAACAAAGTGCTGAAAACCAAATGCCTGCAGGATAACTTTAGC
AAAATTAACAACATTAACAAAATTTTTCTGTGCAGCAGCGGCGGCCCGTTTCAGAACCTGACCATGGATGAACTGAAAAACGTGA
CCAGCGAAAACGCGCTGAAACATCCGAAATGGAAAATGGGCAAAAAAATTACCATTGATAGCGCGACCATGATGAACAAAGGC
CTGGAAGTGATTGAAACCCATTTTCTGTTTGATGTGGATTATAACGATATTGAAGTGATTGTGCATAAAGAATGCATTATTCATAG
CTGCGTGGAATTTATTGATAAAAGCGTGATTAGCCAGATGTATTATCCGGATATGCAGATTCCGATTCTGTATAGCCTGACCTGG
CCGGATCGTATTAAAACCAACCTGAAACCGCTGGATCTGGCGCAGGTGAGCACCCTGACCTTTCATAAACCGAGCCTGGAACAT
TTTCCGTGCATTAAACTGGCGTATCAGGCGGGCATTAAAGGCAACTTTTATCCGACCGTGCTGAACGCGAGCAACGAAATTGCG
AACAACCTGTTTCTGAACAACAAAATTAAATATTTTGATATTAGCAGCATTATTAGCCAGGTGCTGGAAAGCTTTAACAGCCAGAA
AGTGAGCGAAAACAGCGAAGATCTGATGAAACAGATTCTGCAGATTCATAGCTGGGCGAAAGATAAAGCGACCGATATTTATAA
CAAACATAACAGCAGCTGA**

Figure S2. *Pf*DXRt cloned into the pQE30 expression vector. Plasmid map, recombinant protein scheme and amino acid sequence, and DNA sequence of *Pf*DXRt.

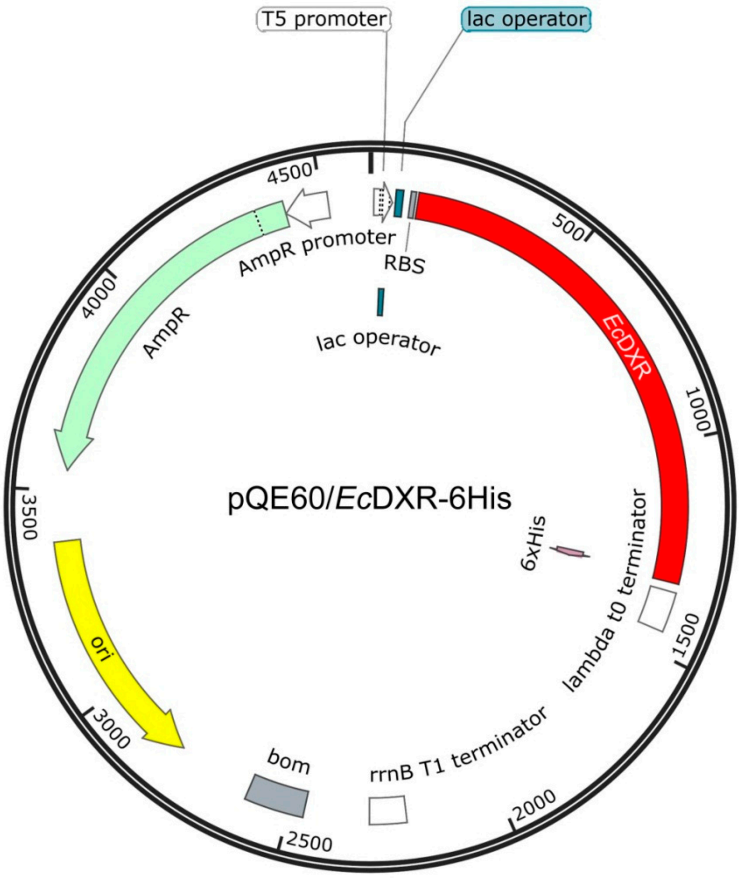

ATG-*Ec*DXR- His tag-stop codon

Recombinant protein sequence (404 amino acids, 44,211 kDa):

**MKQLTILGSTGSIGCSTLDVVRHNPEHFRVVALVAGKNVTRMVEQCLEFSPRYAVMDDEASAKLLKTMLQQQGSRTEVLSGQQAACD
MAALEDVDQVMAAIVGAAGLLPTLAAIRAGKTILLANKESLVTCGRLFMDAVKQSKAQLLPVDSEHNAIFQSLPQPIQHNLGYADLEQN
GVVSILLTGSGGPFRETPLRDLATMTPDQACRHPNWSMGRKISVDSATMMNKGLEYIEARWLFNASASQMEVLIHPQSVIHSMVRYQD
GSVLAQLGEPDMRTPIAHTMAWPNRVNSGVKPLDFCKLSALTFAAPDYDRYPCLKLAMEAFEQGQAATTALNAANEITVAAFLAQQIR
FTDIAALNLSVLEKMDMREPQCVDDVLSVDANAREVARKEVMRLASHHHHHH**

*Ec*DXRt coding sequence (1,212 bases + stop codons):

**ATGAAGCAACTGACCATTCTGGGCAGCACCGGCAGCATCGGTTGCAGCACCCTGGACGTGGTTCGCCATAATCCGGAGCATTTT
CGCGTGGTTGCGCTGGTTGCGGGCAAGAACGTTACCCGTATGGTGGAGCAATGCCTGGAATTCAGCCCGCGTTACGCGGTTATG
GACGATGAGGCGAGCGCGAAGCTGCTGAAAACCATGCTGCAGCAACAGGGTAGCCGTACCGAGGTGCTGAGCGGTCAACAGG
CGGCGTGCGACATGGCGGCGCTGGAAGACGTGGATCAGGTTATGGCGGCGATCGTTGGTGCGGCGGGCCTGCTGCCGACCCT
GGCGGCGATCCGTGCGGGCAAGACCATTCTGCTGGCGAACAAAGAGAGCCTGGTTACCTGCGGTCGTCTGTTCATGGACGCGG
TGAAGCAAAGCAAAGCGCAGCTGCTGCCGGTTGATAGCGAACACAACGCGATCTTTCAAAGCCTGCCGCAACCGATTCAGCAC
AACCTGGGTTATGCGGACCTGGAGCAGAACGGCGTGGTTAGCATTCTGCTGACCGGTAGCGGTGGCCCGTTCCGTGAAACCCC
GCTGCGTGACCTGGCGACCATGACCCCGGATCAGGCGTGCCGTCACCCGAACTGGAGCATGGGCCGTAAGATCAGCGTGGATA
GCGCGACCATGATGAACAAAGGTCTGGAGTACATTGAAGCGCGTTGGCTGTTTAACGCGAGCGCGAGCCAAATGGAAGTGCTG
ATCCACCCGCAGAGCGTTATTCACAGCATGGTGCGTTATCAAGACGGTAGCGTTCTGGCGCAGCTGGGCGAACCGGATATGCGT
ACCCCGATTGCGCACACGATGGCGTGGCCGAACCGTGTTAACAGCGGCGTGAAGCCGCTGGACTTCTGCAAACTGAGCGCGCT
GACCTTTGCGGCGCCGGACTACGATCGTTATCCGTGCCTGAAGCTGGCGATGGAGGCGTTTGAACAAGGTCAAGCGGCGACCA
CCGCGCTGAACGCGGCGAACGAAATTACCGTGGCGGCGTTCCTGGCGCAACAGATCCGTTTTACCGACATTGCGGCGCTGAAC
CTGAGCGTTCTGGAGAAAATGGATATGCGTGAACCGCAGTGCGTGGACGATGTTCTGAGCGTGGATGCGAACGCGCGTGAGGT
TGCGCGTAAAGAGGTTATGCGTCTGGCGAGCCACCACCATCATCATCATTAATGA**

Figure S3. *Ec*DXR cloned into the pQE60 expression vector. Plasmid map, recombinant protein scheme and amino acid sequence, and DNA sequence of *Ec*DXR.

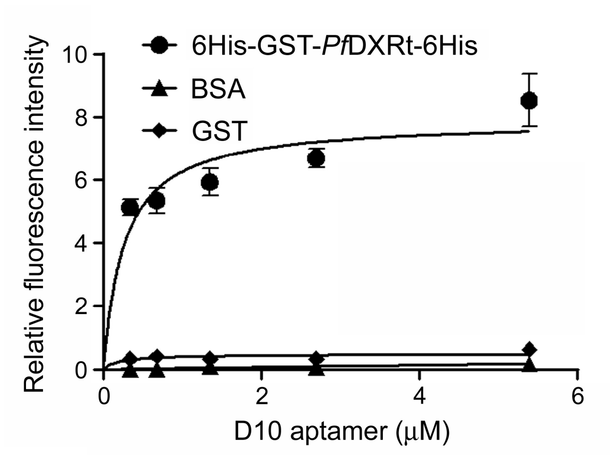

Figure S4. Negative control binding of D10 to free GST. Binding to BSA and to 6His-GST-*Pf*DXRt-6His are included as references.

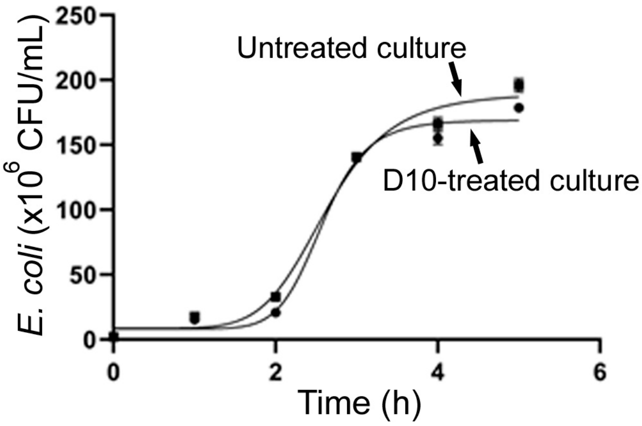

Figure S5. Effect of 50 µM D10 on the growth of an *E. coli* culture.

Table S1. Sequences of 96 subcloned individual oligonucleotides. Sequence 44 corresponds to the D10 aptamer. The number of times that each individual sequence was obtained in the subcloning process is indicated. The PCR primer binding sequences are shown in red.

Code	DNA sequence	Repeats
1	ATACCAGCTTATTCAATTCAGCACATCCACCCACGTGTCCCTCCCGCCACTCGTCGCGAGATAGTAAGTGCAATCT	1
2	ATACCAGCTTATTCAATTGGGCACACACACGGTAGTGTGCCCCCCGCCACGTGGCATCAGATAGTAAGTGCAATCT	1
3	ATACCAGCTTATTCAATTCGCCCACACATACCCCTCTACCGCGTGGCACCATGCATGCAGATAGTAAGTGCAATCT	1
4	ATACCAGCTTATTCAATTGACCCAGTCTGCACATGGGGAGTGTGTGCCGTGCTGCATCAGATAGTAAGTGCAATCT	1
5	ATACCAGCTTATTCAATTCACTGCCACACCACTCCCTGCCATCCGCACATCCATGGTAAGATAGTAAGTGCAATCT	1
6	ATACCAGCTTATTCAATTGACCCAATCAGTACGTGGGGAGCGTGTGCCGTGCTGCAGGAGATAGTAAGTGCAATCT	1
7	ATACCAGCTTATTCAATTGGTGAGCAACATGCACCCACGTGTCCCTGCCCGTCATGCGAGATAGTAAGTGCAATCT	1
8	ATACCAGCTTATTCAATTGCGATGAGTAGTGCACATGACCCGCCACCCTGCTGCATCGAGATAGTAAGTGCAATCT	1
9	ATACCAGCTTATTCAATTCGCGGCACGGGGGGCACACAACGCGGTCGATGCGGCATTCAGATAGTAAGTGCAATCT	1
10	ATACCAGCTTATTCAATTCCGCTCGCCGCAGCCGAACGACCGAGCGCAGCGAGTCAGGAGATAGTAAGTGCAATCT	1
11	ATACCAGCTTATTCAATTACCATGCATACACACCAGTCTGTGCGCGGTCATGTCATGTAGATAGTAAGTGCAATCT	1
12	ATACCAGCTTATTCAATTCCGGTGCGATGTGCAGCTGTCCTGTCAGTCCGTGCGTGTGAGATAGTAAGTGCAATCT	1
13	ATACCAGCTTATTCAATTGCCAACACACCACTGGCCAGGGACACTCGTGATGCGCATCAGATAGTAAGTGCAATCT	1
14	ATACCAGCTTATTCAATTCGACCACATGGCATCACCCACTGTTGTGCATTCATGGCACAGATAGTAAGTGCAATCT	1
15	ATACCAGCTTATTCAATTCGACATGGCGGGCGGAATGCGCGCACGTACGTGTGTGGTGAGATAGTAAGTGCAATCT	1
16	ATACCAGCTTATTCAATTCACTGCCACACAACGCCCTGCCATCCGCACATCCATCGTGAGATAGTAAGTGCAATCT	1
17	ATACCAGCTTATTCAATTGCCGTGTGGCGGGCGGGAGTGCACCAGGTGATGTTGTGTGAGATAGTAAGTGCAATCT	1
18	ATACCAGCTTATTCAATTCAGAAGGGAGCGCCGACACAGGGCGTGGGTGGGCAAGGGAAGATAGTAAGTGCAATCT	1
19	ATACCAGCTTATTCAATTTCCACATGACAGCCCTCACGGTGCGGGGTGCCCGTGGTGAAGATAGTAAGTGCAATCT	1
20	ATACCAGCTTATTCAATTCAACGGAAGAACATGCCGAGGCCGCATCACACCATTCCTGAGATAGTAAGTGCAATCT	1
21	ATACCAGCTTATTCAATTGCACGATCGACATTCAGCGCATGTTCCCCGCACCTACACCAGATAGTAAGTGCAATCT	1
22	ATACCAGCTTATTCAATTGTCCGATGCAACTGCGCCCACACTGTGTCTCCACGCATTCAGATAGTAAGTGCAATCT	1
23	ATACCAGCTTATTCAATTCGCGACGGGACATACGCACCGTGATGGGTGTACATGTGGGAGATAGTAAGTGCAATCT	1
24	ATACCAGCTTATTCAATTGACCCAATCAGTACGTGGGGAGCGTGGGCCGTGCTGCAGGAGATAGTAAGTGCAATCT	1
25	ATACCAGCTTATTCAATTGGTGAGCAACATGCACCCACGTGTGGCTGCCCGTCATGCGAGATAGTAAGTGCAATCT	1
26	ATACCAGCTTATTCAATTCGCATCAAACCGATGTACGCGTGCCCCCTGTTGTTGAGTCAGATAGTAAGTGCAATCT	1
27	ATACCAGCTTATTCAATTCGCGGCACGGGGGGCACACACCGCGGTCGATGCGGCATTCAGATAGTAAGTGCAATCT	1
28	ATACCAGCTTATTCAATTCCGCTCGCCGCAGCCGAACGACCGAGCGCAGCGAGTCAGGAGATAGTAAGTGCAATCT	1
29	ATACCAGCTTATTCAATTACCATGCATACACACCAGTCTCTGCGCGGTCATGTCATGCAGATAGTAAGTGCAATCT	1
30	ATACCAGCTTATTCAATTCCGGTGCGCTGTGCAGCTGTCCTGTCAGTCCGTGCGTGTGAGATAGTAAGTGCAATCT	1
31	ATACCAGCTTATTCAATTCCGACCACATGCCATCACCCACTGTTGGGCATTCATGGCAAGATAGTAAGTGCAATCT	1
32	ATACCAGCTTATTCAATTCGACATGGCGGGCGGCCTGCGCGCACGTACGTGTGTGGTGAGATAGTAAGTGCAATCT	1
33	ATACCAGCTTATTCAATTCACTGGGACACCACGCCCTGCCATCCGCCCATCCATCGTTAGATAGTAAGTGCAATCT	1
34	ATACCAGCTTATTCAATTCGCGACGGGACATACGCAACGTGATGGGTGTACATGTGGTAGATAGTAAGTGCAATCT	2
35	ATACCAGCTTATTCAATTCGTGGGCAGGGAACATCAGCAGTACCAGTGTGTGTGTGGTAGATAGTAAGTGCAATCT	3
36	ATACCAGCTTATTCAATTCGAACCAACGTGATGATGCAACTGTGGCCATGTGGTGGGTAGATAGTAAGTGCAATCT	3
37	ATACCAGCTTATTCAATTGACCCAATCAGTACGTGGGGAGCGTGTGCCGTGCTGCATCAGATAGTAAGTGCAATCT	4
38	ATACCAGCTTATTCAATTCGAACCAACGTGATGATGCAACTGTGGCCATGTGGTGGGTAGATAGTAAGTGCAATCT	4
39	ATACCAGCTTATTCAATTGATACGTACACACCGATCCGGCTGGTTGGCATGTGGCATGAGATAGTAAGTGCAATCT	5
40	ATACCAGCTTATTCAATTCGACATGGCGGGCGGAATGCGCGTACGTACGTGTGTGGTGAGATAGTAAGTGCAATCT	6
41	ATACCAGCTTATTCAATTCGACCATCCATGCCAGCTCGGCCAAGTTGTGGTGTGTGTGAGATAGTAAGTGCAATCT	6
42	ATACCAGCTTATTCAATTCGCATCAAACCGATGTACGCGTGCCACATGTTGTTGAGTCAGATAGTAAGTGCAATCT	6
43	ATACCAGCTTATTCAATTCACGACGGGACATACGCAACGTGATGGGTGTACATGTGGTAGATAGTAAGTGCAATCT	8
D10	ATACCAGCTTATTCAATTCACTGCCACACCACGCCCTGCCATCCGCACATCCATCGTGAGATAGTAAGTGCAATCT	16
